# The three rules of mountaineering and amodal volume completion

**DOI:** 10.1177/20416695251359216

**Published:** 2025-07-24

**Authors:** Vebjørn Ekroll, Rob van Lier

**Affiliations:** 1 Department of Psychosocial Science, 1658 University of Bergen, Norway; 2 Donders Institute for Brain, Cognition and Behaviour, Radboud University, Nijmegen, the Netherlands

**Keywords:** amodal volume completion, perceptual organization, 3D perception, symmetry, seeing-vs-thinking

## Abstract

When climbing a mountain, one is sometimes surprised at how the mountain turns out to be much taller than one initially believed. Wishful thinking easily comes to mind as an explanation for this, but we illustrate how this misjudgment may also be explained as a consequence of the perceptual experience of amodal volume completion.

## How to Cite This Article

Ekroll, V., & van Lier, R. (2025). The three rules of mountaineering and amodal volume completion. *i–Perception*, *16*(4), 1–5. https://doi.org/10.1177/20416695251359216

“It's always further than it looks. It's always taller than it looks. And it's always harder than it looks.” – Anonymous, “The three rules of mountaineering.”

Wishful thinking easily come to mind as an explanation for the presumably quite common experience that inspired the above well-known saying, but as we shall argue in the present note, a perceptual illusion of amodal volume completion (Michotte et al., 1964; Tse, 1999; van Lier & Wagemans, 1999) may also play a significant role.

**Figure 1.**
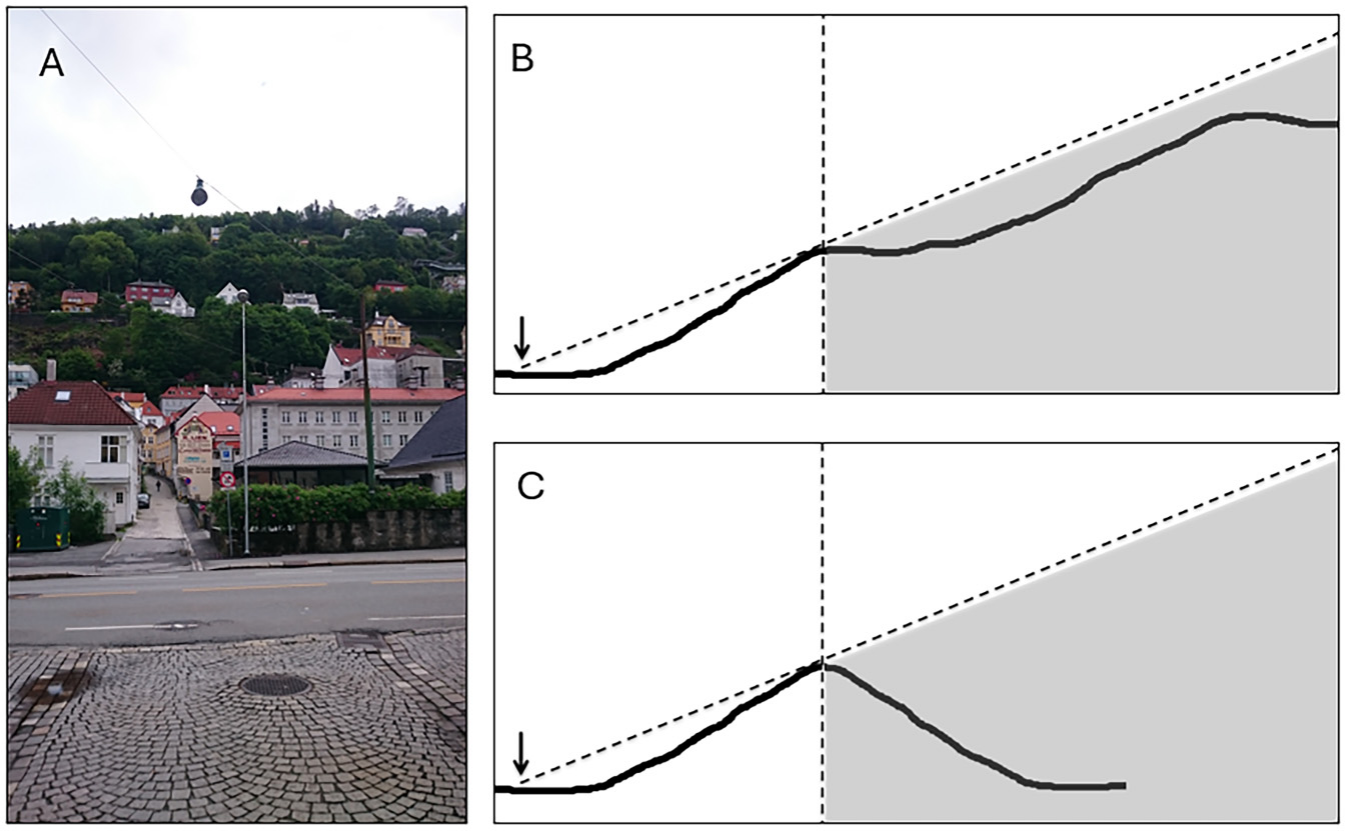
(A) A photograph of Mt. Fløyen, taken from the vantage point of the participants in our study, which is indicated by the arrow in (B). (B) The solid curve shows the height profile of Mt. Fløyen along the line of sight of the participants. Only that part of the height profile which is to the left of the vertical dotted line is visible from the vantage point of the participants. The gray Aaea indicates a region of space that is invisible to the participants due to occlusion. (C) A possible continuation of the visible height profile based on a preference for symmetry.

Consider the photo in Figure 1(A), which is taken at the foot of a mountain.^1^ The height profile of the mountain is shown as a solid curve in Figure 1(B), where the arrow indicates the position from which the photo was taken. Viewed from this position, the part of the mountain in the gray area is invisible due to occlusion by the part of the mountain facing the observer. Thus, based on what the observer can see from this position, the actual height profile of the mountain behind the horizontal dashed line cannot be distinguished from any other height profile contained within the gray area, such as, for instance, the one shown in Figure 1(C). Nevertheless, when looking at this mountainside, we had a distinct feeling that the mountain “looked” more like the symmetric profile in Figure 1(C) than the actual profile in Figure 1(B).

**Figure 2.**
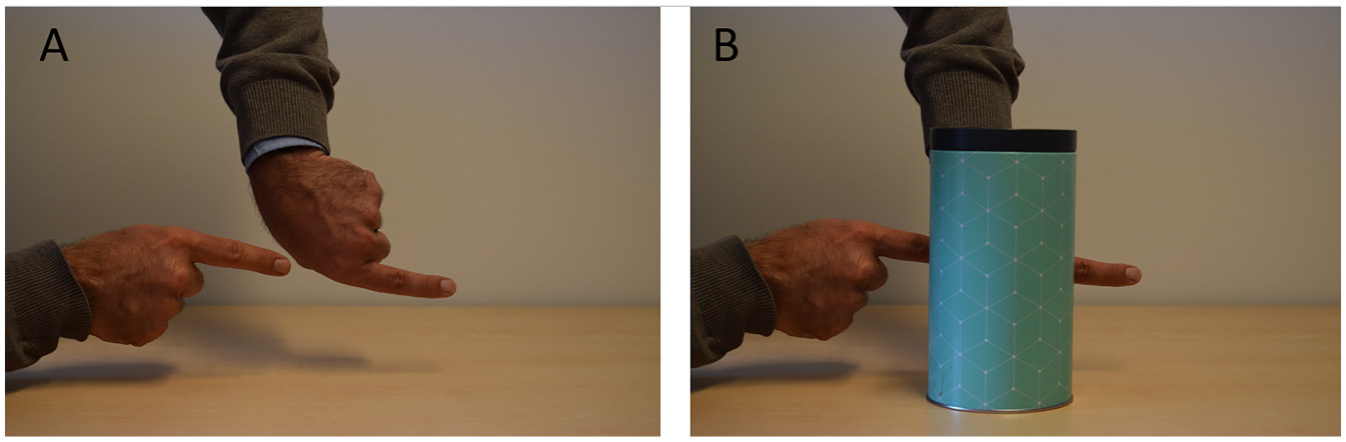
The two index fingers in (A) look like a single long finger in (B) when the gap between them is occluded. In the present experiment, this illustration was used to clarify the potential difference between knowledge and perception to the participants. Adapted from Ekroll et al. (2018, p. 3), used under CC BY.

To find out to what extent other people have experiences similar to ours, we asked 17 passersby to indicate their experiences of the hidden parts of the mountain according to two different criteria by completing the height profile shown in Figure 2(A). After agreeing to participate and providing informed consent, the participants were instructed as follows:

In a moment, we shall ask you to describe your experience of the mountain up there according to two different criteria. In the first round, we will ask you to describe what you believe or can work out by guessing. In the second round, we will ask you to describe your immediate experience.

**Figure 3.**
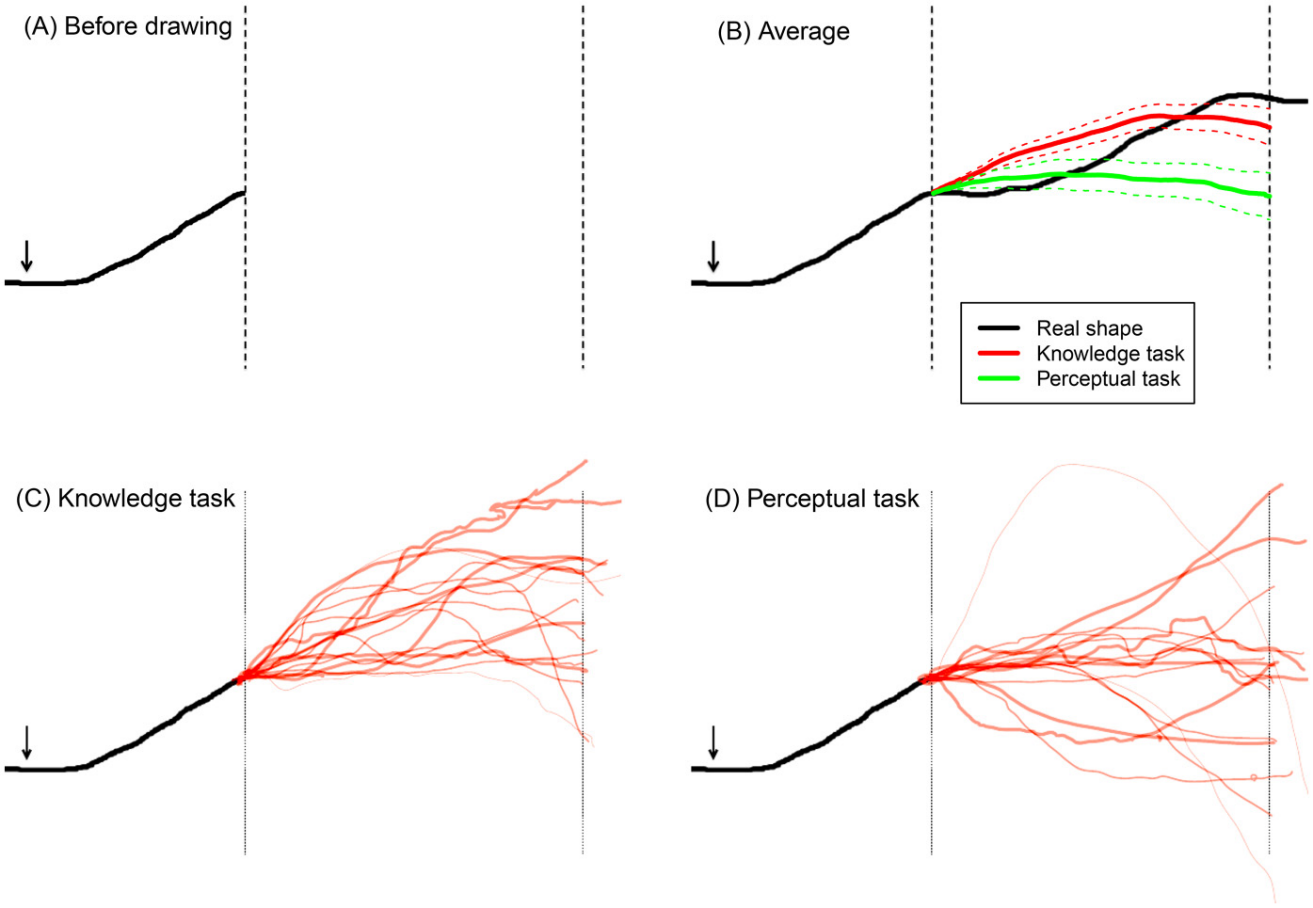
(A) In the experiment, the participants were presented with this sketch of the visible part of the height profile of Mt. Fløyen and asked to complete the invisible part between the two vertical lines according to two different criteria. (B) Averages of the individual curves shown in (C) and (D) overlaid on the real height profile, both with 95% confidence intervals. (C) Individual responses in the knowledge tasks, each of the red curves is from one participant. (D) Individual responses in the perceptual task.

To further clarify the difference between the two criteria, we then showed them the demonstration of amodal completion in Figure 2 and pointed out that when viewing the picture in Figure 2(B), they would probably have the immediate experience of a single, unnaturally long finger, even though there are simply two normal fingers hidden behind the cylinder, as shown in the picture in Figure 2(A). We further explained that “if you are to describe what you believe or know,” you would in this case say “two fingers,” but if you are to describe your immediate experience, you would say “one abnormally long finger.” After this clarification, we commenced by showing the participant the sketch in Figure 3(A) and asking them to perform the “knowledge task” as follows:

Here you see a sketch of the mountain you see in front of you. You are standing at the arrow and face this way towards the mountain [the experimenter points from left to right in the drawing]. The drawing ends at the highest point of the mountain that is visible from here. The part of the mountain behind the first dashed line can’t be seen from here. Please illustrate what you believe the shape of the mountain is like behind the dashed line, based on what you know or can guess. Please start drawing at the first dashed line and end at the second dashed line.

After completing this “knowledge task,” they were asked to complete a “perceptual task” according to the following instruction:

We would now like you to draw the same once more, but this time we would like you to draw your immediate impression of the shape of the mountain independent of what you actually know. Go by the immediate gut feeling you experience when viewing the mountain.

Figure 3(C) shows the results from the knowledge task, while Figure 3(D) shows the results from the perceptual task. In both cases, each of the red curves shown is the drawing made by a single participant. As can be seen, there is considerable individual variation, but on average, the curves drawn in the perceptual task indicated a lower height of the unseen part of the mountain. This can be seen in Figure 3(B), where the averages of the individual curves for each of the two tasks are shown, along with 95% confidence intervals. These average curves were obtained by averaging along the *y*-axis at each pixel value along the horizontal axis. Note also that although both of the average curves underestimate the height of the second peak (located just to the left of the right-hand vertical line), the average curve based on the perceptual task does so to a much larger extent.

Although we obviously do not see the backsides of things in the literal sense, research on amodal volume completion (e.g., Ekroll et al., 2016; Michotte et al., 1964; Tse, 1999; van Lier & Wagemans, 1999) shows that we nevertheless often have very compelling impressions of them, which are shaped by cognitively impenetrable perceptual mechanisms rather than mere imagery or conscious thinking (Firestone & Scholl, 2015; Pylyshyn, 1999). The misjudgment of the mountain may be understood as a further example of how the perceptual system generates representations of the backsides of things, but now in a real-life context where it apparently extends the environmental input by following its own logic.

Early research on amodal completion tended to focus on stimuli with simple and smooth shapes, where the perceptual impressions of the occluded parts of the objects tend to be strikingly compelling and definite (Michotte et al., 1964). When shapes are less smooth and simple, as is the case with the natural object we consider here, the phenomenological impressions may be more fuzzy or indefinite, and hence easier to overlook (de Wit & van Lier, 2002; Ekroll et al., 2017; van Lier, 1999). The key idea here is that thinking or reasoning about the other side of an object always starts with a *perceptually* restricted class (van Lier, 1999). In such cases, where multiple extensions are plausible, perception does not lead to an immediate phenomenology of a single, salient, completion. Instead, the perceptual process becomes apparent by the fact that it initially *constrains* our conscious thinking. Looking at Figure 1(A), for instance, various mountain shapes might be considered, whereas others do not even enter our minds. Just to check on this: did you consider the possibility that there might also be a *third* peak lurking above the second one? There actually is one. From this point of view, the absence of the third peak might be physically wrong, but it still is a vivid perceptual reality.

## Notes

1. The photograph shows Mt Fløyen in Bergen, Norway.
